# Hippocampal CA1/subiculum-prefrontal cortical pathways induce plastic changes of nociceptive responses in cingulate and prelimbic areas

**DOI:** 10.1186/1471-2202-11-100

**Published:** 2010-08-17

**Authors:** Hiroyuki Nakamura, Yoko Katayama, Yoriko Kawakami

**Affiliations:** 1 Department of Physiology, Graduate School of Medicine, Tokyo Women's Medical University, 8-1 Kawada-cho, Shinjuku-ku, Tokyo 162-8666, Japan

## Abstract

**Background:**

Projections from hippocampal CA1-subiculum (CA1/SB) areas to the prefrontal cortex (PFC), which are involved in memory and learning processes, produce long term synaptic plasticity in PFC neurons. We examined modifying effects of these projections on nociceptive responses recorded in the prelimbic and cingulate areas of the PFC.

**Results:**

Extracellular unit discharges evoked by mechanical noxious stimulation delivered to the rat-tail and field potentials evoked by a single stimulus pulse delivered to CA1/SB were recorded in the PFC. High frequency stimulation (HFS, 100 Hz) delivered to CA1/SB, which produced long-term potentiation (LTP) of field potentials, induced long-term enhancement (LTE) of nociceptive responses in 78% of cases, while, conversely, in 22% responses decreased (long-term depression, LTD). These neurons were scattered throughout the cingulate and prelimbic areas. The results obtained for field potentials and nociceptive discharges suggest that CA1/SB-PFC pathways can produce heterosynaptic potentiation in PFC neurons. HFS had no effects on Fos expression in the cingulated cortex. Low frequency stimulation (LFS, 1 Hz, 600 bursts) delivered to the CA1/SB induced LTD of nociceptive discharges in all cases. After recovery from LTD, HFS delivered to CA1/SB had the opposite effect, inducing LTE of nociceptive responses in the same neuron. The bidirectional type of plasticity was evident in these nociceptive responses, as in the homosynaptic plasticity reported previously. Neurons inducing LTD are found mainly in the prelimbic area, in which Fos expression was also shown to be inhibited by LFS. The electrophysiological results closely paralleled those of immunostaining. Our results indicate that CA1/SB-PFC pathways inhibit excitatory pyramidal cell activities in prelimbic areas.

**Conclusion:**

Pressure stimulation (300 g) applied to the rat-tail induced nociceptive responses in the cingulate and prelimbic areas of the PFC, which receives direct pathways from CA1/SB. HFS and LFS delivered to the CA1/SB induced long-term plasticity of nociceptive responses. Thus, CA1/SB-PFC projections modulate the nociceptive responses of PFC neurons.

## Background

The two segregated central pathways for sensory-discriminative and affective dimensions of pain have been examined in human brain imaging studies [1], which indicated that neural activities of the prefrontal cortex (PFC) participate in the affectional dimension of pain [2,3]. Basic lesion studies in animals have implicated the PFC as the center of the pain-related fear emotion [4], which involves affective responses to noxious and fear stimuli [5]. The PFC also has a possible role in the execution and storage of long-term memory [6]. Using PFC slices, high frequency stimulation (HFS) delivered to layer II of the PFC induced long-term depression (LTD) or potentiation (LTP) in neurons of layer V [7]. HFS delivered to CA1 regions induced NMDA (N-Methyl-D-Aspartate)-mediated LTP in the PFC [8], indicating CA1-PFC pathways to be the site of postsynaptic excitatory potentiation.

CA1 pyramidal cells in the hippocampus (HP) receive pain information from peripheral nociceptors [9]. In a rat in vivo study, CA1 pyramidal cells were depressed by intense noxious stimulation applied to the tail [10]. Human brain imaging analysis showed that noxious laser stimulation evoked pain responses in the HP [11]. Responses to noxious heat stimulation in the bilateral HP were markedly increased in an anxious state as compared to circumstances not associated with anxiety [12]. These reports suggest that HP might be involved in the affectional dimension of pain.

The projections from the HP to the PFC were established by anatomical [13,14] and physiological [15] studies. HP projections terminate on spiny pyramidal neurons of the PFC and form excitatory synapses [16]. Excitatory unit responses evoked by CA1/SB stimulation were identified in the prelimbic area [17]. Moreover, approximately 70% of prelimbic interneurons were activated by direct projections from the HP, which induced feed forward inhibition of pyramidal cells [18]. The synapses of hippocampal fibers in the PFC can express different forms of plasticity. The direct excitatory glutamate pathways from the CA1/SB to the PFC [19] are related to NMDA receptor mediated LTP [8]. LTD of field potentials was also induced in HP-PFC pathways by low frequency burst stimulation (LFS) [20]. HP-PFC pathways have been suggested to be required for working memory [21,22]. In an animal behavioral study, bilateral inhibition of the PFC reportedly disrupted spatial working memory [23]. HP-PFC pathways may be related to higher mnemonic functions of pain (e.g. fear conditioned leaning).

We analyzed the effects of CA1/SB inputs into the prelimbic and cingulate areas of the PFC on nociceptive responses evoked by peripheral mechanical noxious stimulation. HFS/LFS delivered to the CA1/SB induced LTP/LTD-like changes in nociceptive responses recorded in the PFC, suggesting the HP-PFC pathway to be involved in affectional memory in pain processing.

## Methods

### Animal preparation

Adult male Wistar rats (280~350 g: Sankyo Laboratory Co, Tokyo, Japan) were used in all experiments. The rats were housed under controlled temperature (25°C) and humidity (40 - 50%) conditions with a 12-h light/dark cycle, and had free access to food and water. Experiments conformed to guidelines issued by the National Institutes of Health for Laboratory Animals and all procedures were approved by the Animal Experimental Committee of Tokyo Women's Medical University. Efforts were made to minimize the number of animals used and their suffering. All rats were anesthetized with a single injection of urethane (1.5 g/kg, i.p.) and mounted in a stereotaxic instrument (Narisige, Tokyo, Japan). Body temperature was maintained at 37 - 38°C using a chemical thermo-mat.

### Recording of extracellular discharges

**Figure 1:**
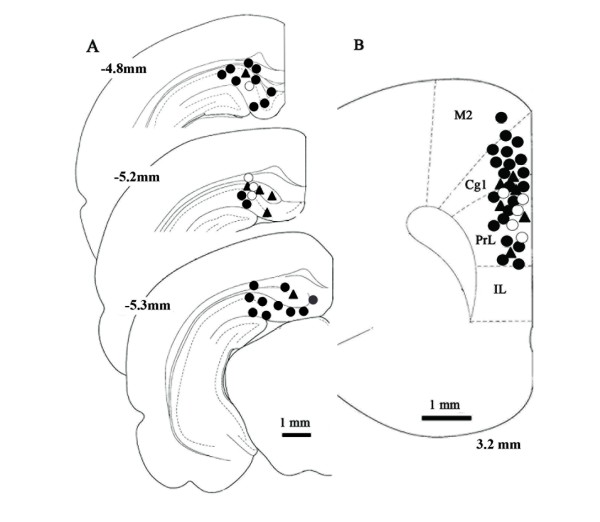
**Locations of stimulus and recording sites**. A: Stimulus locations in CA1/subiculum areas. Numbers on the left represent distance from the bregma. B: Recording sites in the PFC. Solid circles represent the locations of neurons in which LTE was induced by HFS. Triangles represent LTD induced by HFS. Open circles are neurons in which LTD was induced by LFS. The number represents distance from the bregma. M2: secondary motor cortex, Cg1:cingulate cortex area 1, PrL: prelimbic cortex, IL: infralimbic cortex.

**Figure 2:**
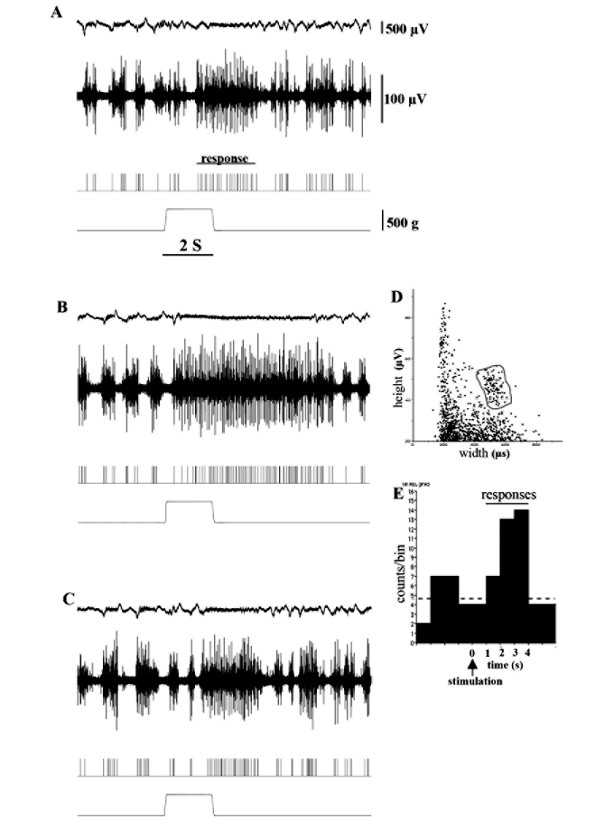
**Effects of CA1/SB inputs on nociceptive responses recorded in the PFC**. A: Nociceptive responses evoked by mechanical stimulation. The top trace represents an electrocorticogram (ECo). The second trace is multiple unit discharges evoked by mechanical stimulation (500 g pressure bottom trace). The third trace represents single unit responses selected by cluster analysis from multi-units in the second line. The bottom trace represents a pressure curve. B: 30 min after HFS delivery to the subiculum areas of the HP, the discharge durations of nociceptive neurons were increased. C: 90 min after HFS nociceptive responses had recovered to the pre-control level. D: Discriminator view obtained using Spike Histogram software. Spikes were selected with the gray line representing single responses. E: Spike histogram composed of the spikes selected above. One bin is 1 s.

Recording electrodes (stainless steel 9 - 12 MΩ, USA) were placed in the cingulate or prelimbic areas of the PFC (coordinates: 3.2 mm anterior and 0.5 mm lateral to the bregma, Fig. 1). The multi or single unit spikes were processed with a multichannel amplifier (MEG-6100; Nihon Koden Co., Tokyo, Japan) and an active filter (500 Hz - 3 KHz, DV-04, NF Electronic Instruments Co.Tokyo, Japan). Data were sent to a personal computer (Macintosh G4; Apple Co., Tokyo, Japan) via an integrated system (Power Lab/4SP; Mountain View, CA, USA) for recording storage and later off-line analysis. A single unit spike was discriminated on the basis of the height and width of each unit from a multi-unit recording (cluster analysis, Fig. 2A~D) obtained with software Chart 4.1 (AD Instruments Co., Tokyo, Japan). Single unit discrimination from multi-units is presented on the third line of Fig 2A~C. The spike histogram was analyzed using clusters of single spikes. Each bin of histograms consists of spikes recorded during a 500 ms or 1000 ms period (Fig 2E).

### Mechanical stimulation

We applied mechanical pressure to the rat-tail, at 1.0 - 2.0 cm distal to the body, using a mechanical stimulator (DPS-270 DIA Medical System Co. Tokyo, Japan), equipped with a probe with a circular contact area and a 1 mm in diameter tip. Mechanical stimuli were delivered every 90 s at constant force with a feedback system. Stimulus intensities used in this experiment were 300 - 500 gf with a 0.1 s rising (and decreasing) time to maximum force and a 2 s hold time. The stimulus condition applied to the tail evoked c-fiber activities in peripheral nerves [24].

### Electrical Simulation

In these experiments, a monopolar stainless steel stimulus electrode (stainless steel 1-3 MΩ, USA) was lowered into the dorsal portion of the CA1/SB area (coordinates: 4.8~5.3 mm posterior and 2.0 - 3.2 mm lateral to the bregma, and 1.8 - 3.6 mm in depth from the surface Fig 1). HFS (100 Hz frequency, 250 μs duration, 20 μA intensity pulse) of 15 sec duration was delivered as a conditioning stimulation (n = 27). LFS was delivered in 600 bursts (1 Hz, 100 μA, 5 pulses). Unit discharges evoked by nociceptive stimulation were recorded every 90 s and the last three data points served as pre-HFS/LFS control values. The stability of responses for at least 20 min before pre-control recording was confirmed.

### Field potential recording in preliminary experiments

**Figure 3:**
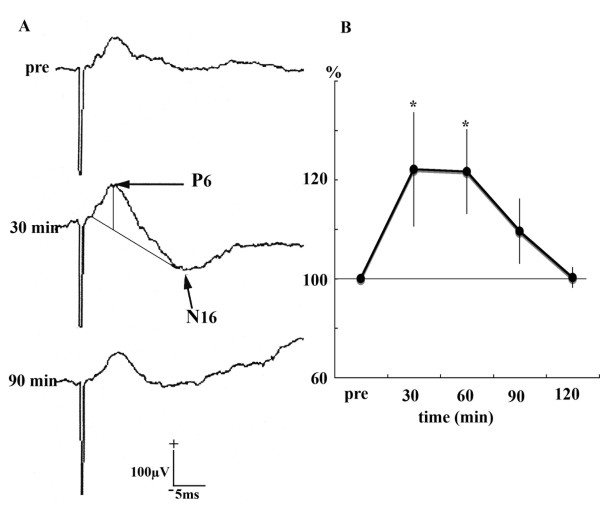
**Field potentials evoked by CA1/subiculum stimulation**. A: A field potential recorded in the PFC was composed of a positive wave with a 6 ms peak latency and a negative wave with a 16 ms peak latency. HFS delivered to CA1/SB increased the amplitude of the positive waves. B: Potentiation of field potentials started within 10 min after HFS and returned to the control value in approximately 90 min.

In the preliminary experiments, we confirmed that the stimulus condition for HFS used in our experiments induced LTP (Fig 3). Field potentials evoked by CA1/SB stimulation (single pulse, 250 μs duration, 100 - 140 μA) were recorded (0.5 Hz - 30 KHz) before and after HFS delivery to the CA1/SB in the PFC. The PFC field potentials evoked by stimulation applied to CA1/SB, which contained 6 ms peak latency positive waves (P6) and 16 ms latency negative waves (N16), were recorded in the PFC (5 animals). LTP of these field potentials was induced by HFS delivered to the CA1/SB and persisted for about 60 min. The maximum increase in amplitude (144.1% ± 23, p < 0.05) was observed at 30 min after HFS, while values at 60, 90 and 120 min were 143 ± 17% (p < 0.05), 119 ± 13% and 100 ± 4%, respectively. The HFS used in our experiments confirmed LTP of field potentials, as demonstrated by previous studies [17,25].

### Locations of unit recording and stimulus electrodes

The locations of units and stimulus points were marked with a positive electric current lesion (direct current, 80 μA for 10 s). At the end of each experiment, the animals were perfused with normal saline and 4% paraformaldehyde. After overnight post-fixation, the brains were sectioned (50 μm) and stained with hematoxylin-eosin solution for examination of the recording and stimulus sites under light microscopy.

We counted the number of Fos-positive cells in the PFC [26] with HFS (5 control animals and 5 animals with HFS) and LFS (5 control animals and 5 animals with LFS). All animals were perfused with 4% paraformaldehyde one hour after conditioning stimulation delivered to the HP, followed by Fos staining. The brains were left in 10% sucrose overnight and then stored frozen. The frozen brain tissues were sectioned at 10 μm (Cryostat CM1850, Leica) and incubated with anti-c-Fos antibody (x 10000, Ab-5, Oncogene, CatnoPC38) overnight at 4°C. Numbers of Fos positive cells in 400 μm^2 ^areas in the cingulate and prelimbic areas were counted in 15 slices for each animal.

### Statistical Analysis

Significant differences in discharges evoked by mechanical stimuli were assessed with the nonparametric paired-test (Wilcoxon) to compare pre- and post-stimulation values. Data are expressed as means ± standard errors (S.E.). Results for the numbers of Fos positive cells were statistically analyzed with the Mann-Whitney test (untreated group versus HFS/LFS group). A probability level of < 0.05 was considered significant.

## Results

### HFS changes pain responses in the PFC

**Figure 4:**
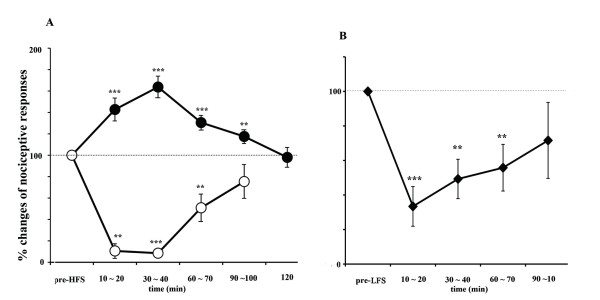
**HFS/LFS induced LTE and LTD of nociceptive responses in the PFC A: HFS (100 Hz, 20 μA, 15 s) delivered to CA1/SB induced LTE (solid circles) or LTD (open circles) of nociceptive responses in the PFC**. B: LFS (1 Hz, 5 pulses, 600 bursts) induced LTD of nociceptive responses (solid squares). The means of three successive trials before HFS (or LFS) served as the control value (100%). Statistical analysis: ** p < 0.01, ***p < 0.001

Noxious mechanical stimulation delivered to peripheral tissue elicited unit discharges and the duration of these responses reportedly reflects stimulus intensity [27]. We measured the duration of noxious discharges to assess the intensity of responses. HFS delivered to the CA1/SB induced two types of long lasting changes in nociceptive responses in the PFC. (Fig 4A). Seventy-eight percent (21/27 animals) of nociceptive neurons showed increases in response duration while, conversely, 22% (6/27 animals) of PFC nociceptive neurons showed decreases of response durations. The plastic changes in nociceptive responses induced by HFS were seen within 10 min after applying HFS and persisted for 120 min. The plasticity processes with maximal effects at 30 ~ 40 min coincided with the field potential changes observed in our preliminary experiments. LTE of pain responses (40 neurons in 21 animals) was 142.7 ± 11% versus the pre-stimulus control at 10 ~ 20 min (p < 0.001), 163.7 ± 10% at 30 ~ 40 min (p < 0.0001), 130.5 ± 7% at 60 ~ 70 min (p < 0.001), 117.5 ± 6.5% at 90 ~ 100 min (p < 0.03) and 97 ± 9.3% at 120 min (p = 0.4). All recording points were located at superficial layers II/III of the cingulate and prelimbic areas (Fig 1). Unit discharges (12 neurons in 6 animals), which showed LTD, occurred mainly in the prelimbic area (Fig 1) and stimulus points were scattered throughout the CA1/SB areas. LTD of pain responses was 10.5 ± 7% at 10 ~20 min (p < 0.02,), 8.5 ± 3 at 30~ 40 min (p < 0.0001), 51 ± 13% at 60 ~ 70 min (p = 0.03) and 75 ± 16% at 90 ~ 100 min (p = 0.17).

### LFS induced LTD of pain responses

LFS delivered to the CA1/SB induced LTD of nociceptive responses (10 neurons in 5 animals, Fig 4B) in the PFC. The onset of decreased pain responses was seen at approximately 250 stimulus bursts and depression gradually reached profound levels. Pain responses decreased to 33.5 ± 11% at 10 ~ 20 min (p < 0.005), 49.3 ± 11% at 30 ~ 40 min (p < 0.01), 71.6 ± 22% at 60 ~ 70 min (p < 0.02) and then recovered to the control level by 90 ~ 100 min (p = 0.3). Background spontaneous discharges decreased markedly in all cases, while, on the contrary, HFS did not affect background activities. All recording points were located at superficial layers II/III of the cingulate and prelimbic areas (Fig 1). LTD recording points were located only in the prelimbic area and the LTE of nociceptive responses were scattered throughout both the cingulate and the prelimbic areas (Fig 1). In two animals (three units), HFS was delivered to the same neurons after the nociceptive response had recovered to the pre-LFS level. After a one-hour observation period to confirm stable responses, HFS applied to CA1/SB produced LTE of nociceptive responses in the same neurons. LTE of nociceptive responses increased to 151% (mean of three neurons) of the control value at 10 min, 230% at 30 min and 150% at 60 min. The potentiation lasted 60 min, i.e. the entire experimental period. The number of neurons was too small to allow statistical analysis, but the tendencies for LTE were very similar in these three neurons.

### Fos expression in the PFC after HFS or LFS

**Figure 5:**
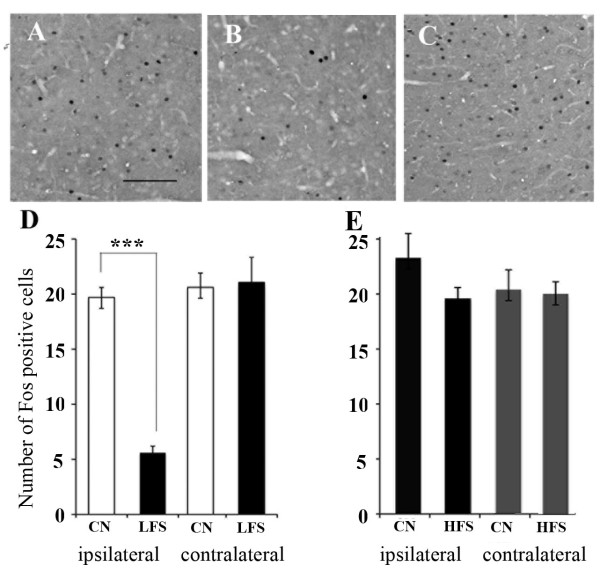
**Fos staining in prelimbic areas of the PFC**. A: An untreated control showed Fos positive cells in the prelimbic area. B: LFS significantly decreased Fos expression in the ipsilateral prelimbic area. C: An untreated control showed Fos positive cells in the cingulate area. Scale lines represent 50 μm. D: Numbers of Fos-expressing cells in the ipsilateral and contralateral prelimbic areas before and after LFS. Fos positive cells were decreased in the prelimbic area. CN: control, LFS: low frequency stimulation *** P < 0.001. E: Numbers of Fos-expressing cells in the ipsilateral and contralateral cingulate areas before and after HFS.

Mean numbers of Fos positive cells in control animals were 23.2 ± 2.2 (left side) and 20.4 ± 1.0 (right side) in the cingulate area and 19.7 ± 0.9 (left side) and 21.9 ± 1.3 (right side) in the prelimbic area (Fig 5). There were no significant differences between the two sides or between the cingulate and prelimbic areas. HFS had no significant effects on Fos expression in the cingulate area (from 23.2 ± 2.2 to 19.6 ± 1), although HFS induced LTE of nociceptive responses. LFS significantly decreased Fos positive cells on the ipsilateral side (5.6 ± 0.6, p < 0.0001, Fig 5) but induced no changes in the contralateral side (23.61 ± 2.3) of the prelimbic area.

## Discussion and Conclusions

Nociceptive information from peripheral tissue mainly projected to the superficial layers of the cingulate and prelimbic areas [27], while subiculum fibers were reportedly distributed in all layers of the prelimbic area [14]. These observations suggest that inputs from the CA1/SB and nociceptive information are integrated in PFC pyramidal cells. In our experiments, HFS delivered to HP/SB induced heterosynaptic potentiation in HP-PFC pathways (reflecting LTP of field potentials) and peripheral-PFC sensory pathways (plasticity of nociceptive responses). The HFS induced either LTE (78%) or LTD (22%) of nociceptive responses. Intracellular recording of PFC neurons demonstrated direct monosynaptic projections from the HP to prelimbic pyramidal cells [28], excitations of which were followed by prolonged inhibition after HP stimulation. The interneurons of the prelimbic area also receive direct projections from the HP [18]. HFS applied to HP could produce synaptic potentiation at both pyramidal cells and interneurons, allowing HFS to induce either LTE or LTD.

NMDA mediated plasticity in the prefrontal cortex was recently reviewed [29]. NMDA receptor subtype NR2B was required for LTP of the prefrontal cortex [30], as in the hippocampus. Moreover, NMDA receptor activities were also required for induction of metabotropic glutamate receptor (mGulR) mediated LTD [31] in the perirhinal cortex. PFC neurons showed induction of LTD with NMDA receptor activities and presynaptic stimulation [32]. Postsynaptic mechanisms may have been responsible for the heterosynaptic plasticity observed in our study. In slice studies, moreover, LTP and LTD could be induced under the same stimulus conditions, depending on the dopamine concentration [33]. Postsynaptic dopamine receptor D1 activation reportedly enhances NMDA mediated-excitatory post-synaptic currents [34] and increases surface expression of NMDA receptors in pyramidal cells [35]. Our experiments were performed in vivo, such that dopamine levels may have varied. Further experiments are necessary to elucidate the synaptic mechanisms underlying the plasticity of nociceptive responses.

LFS delivered to HP/SB induced LTD in nociceptive responses to peripheral noxious stimuli. LFS (1-5 Hz, 300~600 pulses) produces LTD mediated by the glutamate receptor, NMDA [36] and mGulR [37] in the HP. In HP-PFC pathways, a train of 1 Hz bursts of stimulation applied to CA1/SB regions induced LTD of field potentials [20], while HFS produced LTP. HP-PFC pathways commonly expressed bidirectional synaptic plasticity in response to different stimuli [38]. Our results, obtained from only two animals, also showed bidirectional plasticity of nociceptive discharges. Earlier studies demonstrated homosynaptic bidirectional plasticity in the PFC, while the plasticity of nociceptive responses in our experiments was heterosynaptically induced.

LFS significantly decreased Fos expression in cells in the ipsilateral prelimbic area. The histological data were consistent with the areas in which LTD was recorded in the electrophysiological experiments. LFS delivered to HP/SB may inhibit excitation of prelimbic pyramidal cells via HP-PFC pathways. Thus, LFS decreased Fos expression and induced LTD of nociceptive responses.

The PFC is the center of the affectional dimension of pain and involves memories of fear, which are formed by pain experiences. Strong sensory information from peripheral nerves, such as the effects of amputation-induced LTP on synapses receiving information from sensory nerves, may be the cause of phantom pain [39]. Connections between the HP and PFC have been suggested to participate in learning and memory [40]. We established that the plasticity of nociceptive responses recorded in the PFC was produced by HFS/LFS applied to the CA1/SB. The affectional dimension of pain involves memories of fear, which are formed by pain experiences. Our results suggest HP-PFC pathways to be involved in affectional memory in pain processing.

## Authors' contributions

All three authors participated in the preparation of this manuscript. and approved the final manuscript. The individual contributions of three authors to the manuscript are below.

H Nakamura carried out the electrophysiological studies and statistical analysis.

Y Katayama carried out the immunostaining and statistical analysis of Fos expression.

Y Kawakami conceived the study and coordinated all experiments.
